# Seizures in dogs under primary veterinary care in the United Kingdom: Etiology, diagnostic testing, and clinical management

**DOI:** 10.1111/jvim.15911

**Published:** 2020-10-31

**Authors:** Alexander Erlen, Heidrun Potschka, Holger A. Volk, Carola Sauter‐Louis, Dan G. O'Neill

**Affiliations:** ^1^ Institute of Pharmacology, Toxicology, and Pharmacy Ludwig‐Maximilians‐University Munich Germany; ^2^ Department of Small Animal Medicine and Surgery University of Veterinary Medicine Hannover Hannover Germany; ^3^ Institute of Epidemiology Friedrich‐Loeffler‐Institut Greifswald Germany; ^4^ Pathobiology and Population Health The Royal Veterinary College Hatfield United Kingdom

**Keywords:** canine, epilepsy, first‐opinion, fit, IVETF, risk factor, VetCompass

## Abstract

**Background:**

Although seizures are common in dogs, limited published information is available on the classifications of seizures, diagnostic approaches, or clinical management of seizure‐affected patients in the veterinary primary care setting.

**Objectives:**

Explore seizure etiology, diagnostic testing, and clinical management of seizure‐affected dogs in the primary care veterinary setting.

**Animals:**

A total of 455 553 dogs in VetCompass.

**Methods:**

Cross‐sectional analysis by cohort clinical data.

**Results:**

From 2834 incident seizure cases, we identified 579 (20.5%) dogs with epilepsy based on the International Veterinary Epilepsy Task Force (IVETF) classification system, including 484 (17.1%) with idiopathic epilepsy, 95 (3.4%) with structural epilepsy, and 179 dogs (6.3%) with reactive seizures. In their clinical first opinion records, 245 (8.6%) cases were recorded with epilepsy. Overall, 1415 (49.9%) cases received diagnostic evaluation equivalent to or higher than IVETF Tier 1 diagnostic testing.

Being <12 years of age and being insured were risk factors for receiving IVETF Tier 1 or higher diagnostic evaluation among seizure cases. Anti‐seizure drug (ASD) treatment was not prescribed for 1960/2834 (69.2%) dogs in association with the incident seizure event. Of the remainder, 719 (25.3%) dogs received 1 ASD, whereas 155 (5.5%) an ASD combination.

**Conclusion and clinical importance:**

The differences between seizure classifications in the clinical records and those retrospectively assigned by the researchers support the need for clearer diagnostic guidelines in clinical practice. Insured dogs and dogs <12 years of age were more likely to receive advanced diagnostic evaluation, suggesting that financial and perceived prognostic factors influence case management.

**Abbreviations**
ACVIM: American College of Veterinary Internal Medicine
ASD: anti‐seizure‐drug
CI: confidence interval
CNS: central nervous system
CSF: cerebrospinal fluid
DYAR: dog years at risk
EPR: electronic patient record
IQR: interquartile range
IVETF: International Veterinary Epilepsy Task Force
MRI: magnetic resonance imaging
OR: odds ratio
PMS: practice management system
ROC: receiver operating characteristic
RVC: Royal Veterinary College; UK, United Kingdom

## 1 INTRODUCTION

Seizures are common in veterinary practice, affecting approximately 1 in 125 dogs annually under first opinion veterinary care.^1^ Epileptic seizures are described by the International Veterinary Epilepsy Task Force (IVETF) as sudden, short‐lasting, and transient events characterized by motor, autonomic, or behavioral features, or some combination of these, which can be secondary to idiopathic epilepsy (IE), structural epilepsy, or epilepsy of unknown cause. Epilepsy is defined as ≥2 unprovoked epileptic seizures separated by a minimum of 24 hours.^2^ Idiopathic epilepsy is sub‐classified by IVETF into 3 types: genetic (demonstrated genetic background), suspected genetic (evidence of high breed prevalence or familial association), and epilepsy of unknown origin (unidentified underlying cause and absence of structural epilepsy).^2^ Structural epilepsy is characterized by the presence of underlying cerebral or intracranial pathology.^2^ Reactive seizures are not considered as epilepsy but responses of the normal brain to a transient disturbance in function (ie, metabolic or toxic) that is reversible when the cause or disturbance is rectified.^2^, ^3^

Idiopathic epilepsy is a diagnosis of exclusion^4^; IVETF defines 3 levels of diagnostic evaluation (Tiers 1‐3) requiring progressively more advanced diagnostic criteria offering increasing diagnostic confidence.^4^ Tier 1 confidence level includes unremarkable clinical and neurological examinations accompanied by minimum database blood test results and urinalysis. In addition to factors listed in Tier 1, Tier 2 requires unremarkable fasting and postprandial bile acid measurements, magnetic resonance imaging (MRI), and cerebrospinal fluid (CSF) analysis. A Tier 3 diagnostic evaluation includes all components of Tier 1 and 2 plus identification of ictal or inter‐ictal electroencephalographic (EEG) abnormalities.^4^ The probability of first opinion caseloads achieving Tier 1 or higher diagnostic evaluation can be affected by factors including financial constraints, signalment, clinical, and other pet‐related factors, owner compliance, veterinarian clinical skills, practice facilities, and predicted outcomes.^5^

First‐opinion clinical management for seizure cases varies between the needs of immediate emergency care compared with longer‐term non‐emergency management. Emergency care aims to decrease or halt current seizure activity, prevent or decrease further seizures, manage underlying seizure causes, and limit acute complications associated with the seizure event as well as decreasing long‐term consequences.^6^ Non‐emergency longer term care aims to decrease seizure frequency, severity, and duration, but this goal must be balanced with acceptable adverse effects.^7^

We aimed to explore seizure etiology, diagnostic testing, and management in the general population of dogs under primary veterinary care in the United Kingdom (UK). Specific objectives were to describe the demography, seizure classification, diagnostic testing, and treatment both overall and also for the subset of seizure cases specifically recorded as epilepsy in the clinical notes. Further objectives were to evaluate risk factors for receiving at least an equivalent of IVETF Tier 1 or higher diagnostic evaluation and risk factors for a recorded clinical diagnosis of epilepsy with special focus on breed effects.

## 2 MATERIALS AND METHODS

We collected data within the VetCompass Programme^8^ at the Royal Veterinary College (RVC). VetCompass collates electronic patient record (EPR) data from UK primary care practices for epidemiological research. Clinical data from participating practices are extracted from practice management systems (PMSs) by automated queries, uploaded securely to the RVC server and reformatted for entry into the VetCompass online database system.^9^

The study population comprised all dogs in VetCompass with at least 1 EPR (clinical note, bodyweight, or treatment) recorded during 2013 and dogs with at least 1 EPR before and 1 EPR after 2013 or both. These dogs were defined as being under veterinary care during 2013. The year 2013 was selected because it was the latest year of full data available at the time of the study.

A cross‐sectional analysis of cohort clinical data was used for risk factor analysis. A seizure was defined as an event with occurrence of signs characterized by short episodes with convulsive or focal motor, autonomic or behavioral features.^2^ A seizure case was defined as any dog with at least 1 seizure event recorded in the EPR as occurring during 2013. Because the subset of seizure cases specifically diagnosed with epilepsy was of particular interest, descriptive results additionally were reported for the seizure cases recorded in the first opinion clinical notes with epilepsy. Case finding from the overall VetCompass study was as described previously.^1^ The EPRs of confirmed seizure cases from the previous study were evaluated and classified as pre‐existing seizure cases (≥1 seizure recorded before 2013) and “incident seizure cases” (no seizure recorded before 2013). Subsequent analyses included only incident seizure cases. The EPR of each incident seizure case was examined to extract whether or not a clinical diagnosis of epilepsy was recorded by the originating practice. Incident seizure cases additionally were retrospectively categorized based on information recorded in the EPR according to retrospective IVETF classification and diagnostic evaluation. Etiological information in the EPR was used to categorize each seizure case into 1 of 5 categories based on the IVETF classification guidelines^2^: “idiopathic epilepsy,” “structural epilepsy,” “reactive seizures,” “unclassified,” or “no cause recorded.”

For retrospective classification, dogs were required to have evidence of at least 2 seizures separated by a 24‐hour time window for classification as epilepsy.

Data extraction and comparison to IVETF guidelines was performed by the main author (AE), who is a practicing veterinary surgeon. The extraction and classification processes were developed with the support of an expert team of veterinary neurologists and epidemiologists.

Idiopathic epilepsy was defined as dogs between 6 months and 6 years of age at first seizure that had received a minimum of an unremarkable diagnostic evaluation (clinical and neurological examination, basic diagnostic laboratory testing with investigation of CBC, serum biochemistry profile, and urinalysis (including specific gravity, protein, glucose, pH, and sediment evaluation) and for which no alternative seizure cause was recorded (IVETF diagnostic Tier 1).^4^ Structural epilepsy cases were defined as dogs that had interictal neurological deficits, suspected or confirmed structural brain pathology such as neoplasm, inflammation, infection, or degenerative brain lesion, or both reported in the clinical records. Confirmed structural brain pathology required recorded evidence from neuroimaging, CSF analysis or both in the EPR whereas suspected structural epilepsy cases did not require evidence of neuroimaging, CSF analysis or both. Reactive seizure cases had clinical records that described an underlying, reversible seizure cause that was considered a reaction of the normal brain to a transient insult (eg, intoxication, metabolic disorders, electrolyte imbalance, organ dysfunction). Animals were grouped as “unclassified” if the EPR included multiple underlying causes and the animal could not be confidently categorized into any of the first 3 categories. “No cause recorded” included all remaining dogs with no information recorded on seizure causes.

Diagnostic processes recorded in the EPR were extracted and compared to IVETF standards. Cases were classified into 2 groups based on diagnostic evaluation: below IVETF Tier 1 or “IVETF Tier 1 or higher” standard.^4^

Information on MRI or CSF diagnostic evaluation was extracted for each seizure case and for medications prescribed within 48 hours after the first seizure in 2013. Drugs included were diazepam, midazolam, alprazolam, zonisamide, phenobarbital, imepitoin, potassium bromide, gabapentin, levetiracetam, pregabaline, propofol, and medetomidine. The anti‐seizure drug (ASD) regimens were categorized into 3 groups: no medication, single medication or drug combinations. The ASD regimens were reported overall and also by etiological group and clinically diagnosed epilepsy and IVETF diagnostic evaluation classifications.

The “breeds” variable included individual purebred or designer breed types with ≥12 animals presenting as incident seizure cases during 2013. A designer breed type was defined as any dog with a recorded description that was a contraction of at least 2 purebred breed names. A crossbred was defined as any dog with a recorded description that was neither purebred nor designer breed. All remaining purebred and designer breed types were grouped as “other purebreds and designers breed types” and a general category of crossbred dogs also was included. A “purebred ‘variable classified all dogs of a recognizable breed as’ purebred,” all dogs recorded with a designer breed name as “designer”^10^ and all remaining dogs as “crossbred”. A “KC breed group” variable described the UK Kennel Club breed groups. The mean (SD) was used to summarize data that were normally distributed whereas the median (interquartile range [IQR]) was used to summarize non‐normally distributed data.^11^ The age for all incident seizure cases described the age at the first seizure in 2013. An age (years) variable “age at first seizure” contained 7 age categories (0.00‐≤0.50, 0.50‐≤3.00, 3.00‐≤6.00, 6.00‐≤9.00, 9.00‐≤12.00, >12.00 years, unrecorded) in accordance with IVETF protocols.^4^ “Neuter status” recorded the neuter status at the final EPR and was combined with sex into a single variable “Sex‐neuter.” Adult was defined as >18 months of age. “adult body weight” categorized the median adult body weight (kg) into 6 groups (<10.00, 10.00‐≤20.00, 20.00‐≤30.00, 30.00‐≤40.00, ≥40.00, unrecorded). An “adult body weight relative to breed and sex mean” variable characterized the adult body weight of individual dogs as either below or equal to or above the mean adult body weight for their breed and sex within the overall study population. This variable allowed the effect of adult body weight to be assessed within each breed and sex combination. The variable “insurance status” described whether the dog was insured, not insured or unrecorded at the final available EPR. An overview of different variables is presented in Table 1.

**TABLE 1:** Descriptive statistics of incident seizure cases under primary veterinary care in the UK dog population; retrospective IVETF classification

			Retrospective IVETF classification				
		All cases no. (%)	Idiopathic epilepsy no. (%)	Structural epileptic no. (%)	Reactive seizure no. (%)	Unclassified no. (%)	No cause recorded no. (%)
Variable	Category	2834 (100.0)	484 (17.1)	95 (3.4)	179 (6.3)	910 (32.1)	1166 (41.1)
Purebred	Purebred	2240 (79.0)	389 (80.4)	75 (78.9)	144 (80.4)	718 (78.9)	914 (78.4)
	Crossbred	517 (18.2)	79 (16.3)	20 (21.1)	31 (17.3)	164 (18.0)	223 (19.1)
	Designer	68 (2.4)	16 (3.3)	0 (0.0)	4 (2.2)	26 (2.9)	22 (1.9)
	Unknown	9 (0.3)	0 (0.0)	0 (0.0)	0 (0.0)	2 (0.2)	7 (0.6)
KC breed group	Not KC recognized	820 (28.9)	131 (27.1)	27 (28.4)	46 (25.7)	268 (29.5)	348 (29.8)
	Gundog	455 (16.1)	99 (20.5)	10 (10.5)	22 (12.3)	159 (17.5)	165 (14.2)
	Hound	94 (3.3)	21 (4.3)	1 (1.1)	7 (3.9)	29 (3.2)	36 (3.1)
	Pastoral	241 (8.5)	57 (11.8)	8 (8.4)	12 (6.7)	71 (7.8)	93 (8.0)
	Terrier	363 (12.8)	56 (11.6)	17 (17.9)	19 (10.6)	116 (12.7)	155 (13.3)
	Toy	484 (17.1)	78 (16.1)	16 (16.8)	43 (24.0)	149 (16.4)	198 (17.0)
	Utility	235 (8.3)	23 (4.8)	8 (8.4)	18 (10.1)	73 (8.0)	113 (9.7)
	Working	142 (5.0)	19 (3.9)	8 (8.4)	12 (6.7)	45 (4.9)	58 (5.0)
Adult body weight (kg)	<10.00	673 (23.7)	113 (23.3)	17 (17.9)	58 (32.4)	206 (22.6)	279 (23.9)
	10.00‐≤20.00	449 (15.8)	103 (21.3)	21 (22.1)	19 (10.6)	128 (14.1)	178 (15.3)
	20.00‐≤30.00	348 (12.3)	86 (17.8)	7 (7.4)	11 (6.1)	117 (12.9)	127 (10.9)
	30.00‐≤40.00	240 (8.5)	54 (11.2)	6 (6.3)	15 (8.4)	61 (6.7)	104 (8.9)
	≥40.00	77 (2.7)	12 (2.5)	5 (5.3)	5 (2.8)	34 (3.7)	21 (1.8)
	Unrecorded	1047 (36.9)	116 (24.0)	39 (41.1)	71 (39.7)	364 (40.0)	457 (39.2)
		Median (IQR)	Median (IQR)	Median (IQR)	Median (IQR)	Median (IQR)	Median (IQR)
	Median adult bodyweight (kg)	16.12 (8.33–28.04)	19.03 (9.34–29.31)	16.15 (9.00–28.50)	10.80 (6.65–27.86)	16.01 (8.43‐28.58)	15.34 (7.89‐26.56)
Adult body weight relative to breed and sex mean	Lower	1265 (44.6)	211 (43.6)	41 (43.2)	78 (43.6)	435 (47.8)	500 (42.9)
	Equal/Higher	1129 (39.8)	249 (51.4)	36 (37.9)	56 (31.3)	360 (39.6)	428 (36.7)
	Unrecorded	440 (15.5)	24 (5.0)	18 (18.9)	45 (25.1)	115 (12.6)	238 (20.4)
Age at first Seizure (years)	0.00‐≤0.50	91 (3.2)	4 (0.8)	3 (3.2)	18 (10.1)	26 (2.9)	40 (3.4)
	0.50‐≤3.00	622 (21.9)	229 (47.3)	6 (6.3)	30 (16.8)	188 (20.7)	169 (14.5)
	3.00‐≤6.00	728 (25.7)	251 (51.9)	8 (8.4)	31 (17.3)	203 (22.3)	235 (20.2)
	6.00‐≤9.00	533 (18.8)	0 (0.0)	18 (18.9)	28 (15.6)	230 (25.3)	257 (22.0)
	9.00‐≤12.00	397 (14.0)	0 (0.0)	31 (32.6)	37 (20.7)	141 (15.5)	188 (16.1)
	≥12.00	444 (15.7)	0 (0.0)	29 (30.5)	31 (17.3)	116 (12.7)	268 (23.0)
	Unrecorded	19 (0.7)	0 (0.0)	0 (0.0)	4 (2.2)	6 (0.7)	9 (0.8)
		Median (IQR)	Median (IQR)	Median (IQR)	Median (IQR)	Median (IQR)	Median (IQR)
	Median age at first seizure	6.00 (3.00–10.10)	3.15 (2.15–4.40)	11.00 (7.75–13.20)	6.80 (3.00–11.30)	6.50 (3.25‐9.70)	7.70 (4.00‐11.90)
Sex	Female	1240 (43.8)	152 (31.4)	47 (49.5)	103 (57.5)	392 (43.1)	546 (46.8)
	Male	1587 (56.0)	331 (68.4)	48 (50.5)	76 (42.5)	517 (56.8)	615 (52.7)
	Unrecorded	7 (0.2)	1 (0.2)	0 (0.0)	0 (0.0)	1 (0.1)	5 (0.4)
Neuter status	Entire	891 (31.4)	149 (30.8)	29 (30.5)	63 (35.2)	271 (29.8)	379 (32.5)
	Neutered	1303 (46.0)	262 (54.1)	46 (48.4)	78 (43.6)	403 (44.3)	514 (44.1)
	Unknown	640 (22.6)	73 (15.1)	20 (21.1)	38 (21.2)	236 (25.9)	273 (23.4)
Sex‐neuter	Female/Entire	335 (11.8)	37 (7.6)	11 (11.6)	31 (17.3)	103 (11.3)	153 (13.1)
	Female/Neutered	625 (22.1)	96 (19.8)	24 (25.3)	51 (28.5)	184 (20.2)	270 (23.2)
	Female/unrecorded	280 (9.9)	19 (3.9)	12 (12.6)	21 (11.7)	105 (11.5)	123 (10.5)
	Male/Entire	550 (19.4)	111 (22.9)	18 (18.9)	32 (17.9)	167 (18.4)	222 (19.0)
	Male/Neutered	677 (23.9)	166 (34.3)	22 (23.2)	27 (15.1)	219 (24.1)	243 (20.8)
	Male/unrecorded	360 (12.7)	54 (11.2)	8 (8.4)	17 (9.5)	131 (14.4)	150 (12.9)
	Unrecorded/unrecorded	7 (0.2)	1 (0.2)	0 (0.0)	0 (0.0)	1 (0.1)	5 (0.4)
Insurance status	Insured	521 (18.4)	137 (28.3)	13 (13.7)	28 (15.6)	190 (20.9)	153 (13.1)
	Not insured	217 (7.7)	26 (5.4)	7 (7.4)	11 (6.1)	81 (8.9)	92 (7.9)
	Unrecorded	2096 (74.0)	321 (66.3)	75 (78.9)	140 (78.2)	639 (70.2)	921 (79.0)

*Note*: Descriptive statistic results among incident seizure cases in the UK dog population under primary veterinary care; etiologic classification, clinical diagnosis with epilepsy in first opinion setting and diagnostic evaluation.

Abbreviations: IQR, interquartile range; IVETF, International Veterinary Epilepsy Task Force.

After data checking and cleaning in Excel (Microsoft Office Excel 2013, Microsoft Corp.), statistical analyses were conducted by IBM SPSS Statistics 24. The confidence interval (CI) estimates were derived from standard errors, based on approximation to the binomial distribution.^11^ Descriptive statistics were generated to report the incident seizure cases according to: (a) retrospective IVETF classification, (b) clinical diagnosis of epilepsy in the first opinion setting, and (c) diagnostic evaluation.

Binary logistic regression modeling was used to separately evaluate risk factors for an outcome of either receiving an IVETF Tier 1 or higher diagnostic evaluation or a recorded clinical diagnosis with epilepsy in the EPR. For each modeling process, initial univariable evaluation considered the following risk factors: purebred, breed, Kennel Club breed group, adult body weight (categories), adult body weight relative to breed and sex mean, age at first seizure, sex, neuter status, sex‐neuter, and insurance status. Modeling for an outcome of an epilepsy diagnosis in the clinical notes included whether MRI or CSF analysis was performed as additional risk factors. Risk factors with liberal associations in univariable modeling (*P* < .2) were taken forward for multivariable logistic regression modeling evaluation. Breed was a factor of primary interest. Purebred status and Kennel Club breed group were derived by categorizing the breed variable and were therefore highly correlated with breed. Adult body weight is a defining characteristic of breeds. For these reasons, purebred status, Kennel Club breed group and adult body weight were excluded from breed multivariable modeling. Model development used automated backwards stepwise elimination. The area under the receiver operator characteristic (ROC) curve was used to assess the quality of the model fit.^12^ Statistical significance was set at *P* < .05.

## 3 RESULTS

The study population consisted of 455 553 dogs attending primary care practices in the UK participating in VetCompass. Of the study dogs with known sex and neuter status, 100 996/184906 (54.6%) females and 103 902/196325 (52.9%) males were neutered.^1^ Overall, 3731 (35.25%) dogs met the inclusion criteria giving an overall 1‐year period prevalence of 0.82% (95% CI, 0.79‐0.84) for dogs having at least 1 seizure during 2013 as reported recently.^1^ Among those 3731 dogs with seizures, 2834 (75.96%) were incident cases (first recorded seizure in 2013) giving an overall 1‐year incidence risk of 0.62% (95% CI, 0.60‐0.64). These 2834 incident cases were included in the current study analyses.

The 2834 incident cases included 2240 (79.0%) purebred, 517 (18.2%) crossbred, 68 (2.4%) designer, and 9 (0.3%) dogs with an unknown purebred status. The most common breeds were Labrador Retriever (n = 243, 8.6% of incident cases), Staffordshire Bull Terrier (n = 173, 6.1%), Jack Russell Terrier (n = 165, 5.8%), and Yorkshire Terrier (n = 141, 5.0%). The 3 most represented UK Kennel Club breed groups were “Toy” (n = 484, 17.1%), “Gundog” (n = 455, 16.1%), and “Terrier” (n = 363, 12.8%) groups.

The median adult body weight for incident seizure cases was 16.12 kg (IQR, 8.33‐28.04). The median age at the first seizure was 6.00 years (IQR, 3.00‐10.10). Of the 1587 (56.0%) male incident cases, 677 (23.9%) were neutered. Of the 1240 (43.8%) female incident cases, 625 (22.1%) were neutered. There were 521 (18.4%) cases recorded as insured, 217 (7.7%) as not insured, and 2096 (74.0%) as unrecorded insurance status (Table 1). Additional supporting information are displayed as Supplementary file.

### 3.1 Recorded diagnosis of epilepsy in the clinical notes

There were 245 (8.6%) incident cases with a recorded epilepsy diagnosis in the clinical notes. Of these cases, 185 (75.5%) were purebred. Breeds with at least 10 diagnosed epilepsy cases included the Labrador Retriever (n = 24, 9.8%), Border Collie (n = 19, 7.8%), Jack Russell Terrier (n = 12, 4.9%), and Staffordshire Bull Terrier (n = 10, 4.1%). The most common Kennel Club breed group with epilepsy cases was the Gundog (n = 38, 15.5%). Clinically diagnosed epilepsy cases had a median adult body weight of 17.60 kg (IQR, 9.88‐29.28) and median age at first seizure of 5.75 years (IQR, 3.00‐10.45). There were 133 (54.3%) males of which 61 (24.9%) were neutered (Table 1). Of the 245 clinically diagnosed epilepsy cases, 154 (62.9%) dogs had IVETF Tier 1 or higher diagnostic evaluation. Of those 245 dogs, 34 (13.9%) had MRI, and 23 (9.4%) had CSF analysis.

### 3.2 Retrospective IVETF etiological classification

Retrospective review of the EPRs classified 758 (26.8%) of the 2834 total incident cases to 1 of the IVETF categories whereas the remaining 2076 (73.2%) cases were classified as either “unclassified” or “no cause recorded”. Of the 2834 total incident cases, 484 (17.1%) were classified as idiopathic epilepsy. Of these 484 idiopathic epilepsy cases, 52 (10.7%) were recorded with epilepsy in the clinical records. The remaining 434 (89.3%) cases were not recorded with epilepsy in the clinical notes. The retrospective idiopathic epilepsy cases consisted of 389 (80.4%) purebred dogs. The most common breeds among the retrospective idiopathic epilepsy cases were Labrador Retriever (n = 63, 13.0%), Border Collie (n = 36, 7.4%), Staffordshire Bull Terrier (n = 35, 7.2%), and Jack Russell Terrier (n = 23, 4.8%). The most common breed group was the “Gundog” (n = 99, 20.5%) followed by the “Toy” group (n = 78, 16.1%). The median adult body weight of the idiopathic epilepsy cases was 19.03 kg (IQR, 9.34‐29.31) and the median age at first seizure was 3.15 years (IQR, 2.15‐4.40). Neutered males (n = 166, 34.3%) represented the largest group in the sex‐neuter variable (Table 1).

There were 95/2834 (3.4%) incident cases retrospectively classified as either confirmed or suspected structural epilepsy. Of those 95 cases, 8 (8.4%) cases were recorded with epilepsy in the clinical records. The remaining 87 (91.6%) cases were not recorded with epilepsy in the clinical records. These cases included 75 (78.9%) purebred dogs. The most common breeds among the structural epilepsy cases were Staffordshire Bull Terrier (n = 8, 8.2%), Labrador Retriever (n = 6, 6.3%), Jack Russell Terrier (n = 4, 4.2%), and Yorkshire Terrier (n = 4, 4.2%). The most common KC breed groups were “Terrier” (n = 17, 17.9%) and “Toy” (n = 16, 16.8%). The median adult body weight was 16.15 kg (IQR, 9.00‐28.50) and median age at first seizure was 11.00 years (IQR, 7.75‐13.20). There were 31 (32.6%) animals in this group between 9.00 and 12.00 years old and 29 (30.5%) were >12.00 years of age. Within the sex‐neuter variable, 24 (25.3%) dogs were neutered females and 22 (23.2%) were neutered males.

There were 179/2834 (6.3%) incident cases retrospectively categorized as reactive seizure cases. Of those 179 cases, 14 (7.8%) cases were recorded with epilepsy within the clinical records. The remaining 165 (92.2%) cases were not recorded with epilepsy in the clinical records. These consisted of 144 (80.4%) purebred dogs. The most common breeds among the reactive seizure cases were Yorkshire Terrier (n = 14, 7.8%), Labrador Retriever (n = 13, 7.3%), Chihuahua (n = 11, 6.1%), and Jack Russell Terrier (n = 9, 5.0%). The most common breed group was “Toy” (n = 43, 24.0%). The median adult body weight was 10.80 kg (IQR, 6.65‐27.86), and 58 (32.4%) dogs weighed <10.00 kg. Median age at first seizure was 6.80 years (IQR, 3.00‐11.30). Of the reactive seizure cases, 51 (28.5%) were neutered female dogs (Table 1).

### 3.3 IVETF diagnostic evaluation

There were 1415/2834 (49.9%) incident cases with a diagnostic evaluation that achieved IVETF Tier 1 or higher. Of the 245 incident cases diagnosed with epilepsy in the primary care setting, 81 (33.1%) cases received a less than IVETF Tier 1 diagnostic evaluation. Of these 1415 dogs, 1130 (79.9%) were purebred. The most common breeds were Labrador Retriever (n = 136, 9.6%), Staffordshire Bull Terrier (n = 95, 6.7%), Jack Russell Terrier (n = 80, 5.7%), and Border Collie (n = 73, 5.2%). The most common KC breed groups were “Gundog” (n = 240, 17.0%), “Toy” (n = 233, 16.5%), and “Terrier” (n = 201, 14.2%). The median adult body weight for dogs with IVETF Tier 1 or higher diagnostic evaluation was 16.88 kg (IQR, 8.88‐28.84) compared with 15.23 kg (IQR, 7.80‐26.86) for dogs that did not reach IVETF Tier 1 diagnostic evaluation (Mann‐Whitney *U* test, *P* = .13). Median age at first seizure for dogs with IVETF Tier 1 or higher diagnostic evaluation was 5.10 years (IQR, 2.80‐8.90) compared with 6.60 years (IQR, 3.50‐8.90) for dogs that did not reach IVETF Tier 1 diagnostic evaluation (Mann‐Whitney *U* test, *P* < .001).

There were 320 dogs (22.6%) with IVETF Tier 1 or higher diagnostic evaluation that were insured compared with 175 of dogs (14.1%) that did not reach IVETF Tier 1 diagnostic evaluation (chi‐squared, *P* < .001).

### 3.4 Risk factors for recorded clinical diagnosis with epilepsy

Final multivariable logistic regression modeling identified 3 risk factors significantly associated with a recorded epilepsy diagnosis in the clinical notes. These included: IVETF Tier 1 or higher diagnostic evaluation, MRI, and CSF analysis. Animals that received IVETF Tier 1 or higher diagnostic evaluation had 1.74 times the odds (95% CI, 1.30‐2.34; *P* < .001) of clinical epilepsy diagnosis compared to animals that did not reach an IVETF Tier 1 diagnostic evaluation. Animals that underwent an MRI had 6.19 times the odds (95% CI, 3.03‐12.64; *P* < .001) of epilepsy diagnosis compared to animals that did not undergo MRI. Dogs with CSF analysis had 2.80 times the odds (95%, CI 1.13‐6.94; *P* = .03) of epilepsy diagnosis compared to dogs that did not have CSF analysis (Table 2).

**TABLE 2:** Final breed multivariable logistic regression results for risk factors associated with clinical diagnosis with epilepsy in first opinion setting from all incident seizure cases under primary veterinary care in the UK dog population

Variable	Category	Odds ratio	95% CIa		Category *P*‐value	Variable *P*‐value
IVETF Tier 1	Below IVETF Tier 1	Base				*P* < .001
	IVETF Tier 1or higher	1.74	1.30	2.34	<.001	
	Unrecorded	0.89	0.44	1.78	.73	
MRI	No MRI	Base				*P* < .001
	MRI	6.19	3.03	12.64	<.001	
CSF	No CSF	Base				*P* < .001
	CSF	2.80	1.13	6.94	0.03	

*Note*: Risk factors for clinical diagnosis with epilepsy in first opinion setting from incident seizure cases under primary veterinary care in the UK dog population; Base = comparison group.

Abbreviations: CSF, cerebrospinal fluid; IVETF, International Veterinary Epilepsy Task Force; MRI, magnetic resonance imaging.

### 3.5 Risk factors for IVETF Tier 1 or higher diagnostic work‐up

Multivariable logistic regression modeling identified 5 risk factors significantly associated with IVETF Tier 1 or higher diagnostic evaluation: Adult body weight relative to breed and sex mean, age at first seizure, insurance status, clinically recorded with epilepsy, and Kennel Club breed group.

Dogs with an adult body weight equal to or higher than their breed and sex mean had 1.37 times the odds (95% CI 1.15‐1.63; *P* < .001) of receiving IVETF Tier 1 or higher diagnostic evaluation compared to dogs with a lower adult body weight than their breed and sex mean. Compared to animals ≥12 years of age, all age categories for dogs <12 years had significantly increased odds for receiving IVETF Tier 1 or higher diagnostic evaluation. Insured animals had 1.50 times the odds (95% CI, 1.06‐2.13; *P* = .02) for receiving IVETF Tier 1 or higher diagnostic evaluation compared to uninsured animals. Dogs with a clinically recorded diagnosis of epilepsy had 1.74 times the odds (95% CI 1.30‐2.32; *P* < .001) for receiving IVETF Tier 1 or higher diagnostic evaluation compared to animals with no recorded diagnosis of epilepsy (Table 3).

**TABLE 3:** Multivariable logistic regressions results: Risk factors for receiving IVETF Tier 1 or higher diagnostic evaluation

Variable Body weight relative to breed mean	Category Lower	Odds ratio Base	95% CIa		Category *P*‐value	Variable *P*‐value *P* < .001
	Equal/Higher	1.37	1.15	1.63	<.001	
	Unrecorded	0.72	0.55	0.94	.02	
Age at first seizure (years)	0.00‐≤0.50	2.50	1.50	4.18	<.001	*P* < .001
	0.50‐≤3.00	2.87	2.16	3.81	<.001	
	3.00‐≤6.00	2.01	1.54	2.63	<.001	
	6.00‐≤9.00	1.74	1.31	2.31	<.001	
	9.00‐≤12.00	1.67	1.23	2.26	.001	
	≥12.00	Base				
	Unrecorded	2.60	0.90	7.56	.08	
Insurance Status	Is insured	1.50	1.06	2.13	.02	*P* < .001
	Not insured	Base				
	Unrecorded	0.92	0.67	1.28	.63	
Clinically recoded with epilepsy	Not epileptic	Base				*P* < .001
	Epileptic	1.74	1.30	2.32	<.001	

*Note*: Risk factors for receiving an IVETF Tier 1or higher diagnostic evaluation among incident seizure cases under primary veterinary care in the UK dog population; Base = comparison group.

Abbreviation: IVETF, International Veterinary Epilepsy Task Force.

Animals in the UK Kennel Club Terrier breed group had 1.40 times the odds (95% CI, 1.07‐1.83; *P* = .01) of receiving IVETF Tier 1 or higher diagnostic evaluation compared to animals that were not Kennel Club‐recognized.

### 3.6 Medical management

Of 2834 incident seizure cases in 2013, 1960 (69.2%) did not receive any ASD in association with the first seizure event. Of the remaining 874 (30.8%) cases, 719 dogs (25.4%) received a single ASD and 155 (5.5%) an ASD combination in association with the incident 2013 seizure event. Among the cases that were retrospectively classified according to IVETF guidelines, medical treatment was given to 141 (29.1%) dogs with idiopathic epilepsy, 23 (24.2%) with structural epilepsy, 51 (28.5%) with reactive seizure, 358 (30.7%) in which no cause was recorded, and 301 (33.1%) in which the seizure cause could not be classified. Whether dogs with IVETF epilepsy received medical treatment or not did not differ between each IVETF category (*P* = .27). Dogs with seizures that were recorded as having epilepsy in the clinical notes were more likely to receive at least 1 ASD than were dogs with seizures that were not clinically diagnosed as having epilepsy (148; 60.4% vs 726; 28.0%; *P* < .001). The most commonly used drugs overall were benzodiazepine (360; 12.7%), phenobarbital (271; 9.6%), and imepitoin (61; 2.2%). Dogs with clinically diagnosed epilepsy were more likely to receive phenobarbital (64; 25.8%; *P* < .001), an ASD combination (28; 11.4%; *P* < .001), or imepitoin (20; 8.1%; *P* < .001) compared with animals that were not clinically diagnosed with epilepsy. Benzodiazepine use did not differ between dogs that were or were not diagnosed with epilepsy in the clinical notes (*P* = .91). The most commonly used ASD combinations were benzodiazepine and phenobarbital (91; 3.2%) phenobarbital and potassium bromide (18; 0.6%) and benzodiazepine and imepitoin (16; 0.6%). Dogs with clinically diagnosed epilepsy were more likely to receive the ASD combination phenobarbital and potassium bromide (6; 2,5%; *P* < .001) and phenobarbital and potassium bromide and levetiracetam (3; 1.2%; *P* < .001) than were dogs that were not clinically diagnosed with epilepsy.

## 4 DISCUSSION

We report on the etiology, diagnostic evaluation, and clinical management of dogs under primary veterinary care affected with seizures in the UK. Our study of retrospective primary care data identified a seizure incidence risk of 0.62%. Using a slightly different case definition, a Swedish study by pet insurance data identified a similar 0.75% incidence risk for epilepsy in 2014. Both of these retrospective studies used large databases of pre‐existing health information and each was susceptible to different selection biases on the dogs included. The slightly higher epilepsy rates in insured dogs^13^ could reflect higher diagnostic efforts for insured dogs compared to uninsured dogs .

Retrospective classification identified that 20.5% (idiopathic epileptic 17.1% and structural epileptic 3.4%) of the incident seizure cases met the IVETF criteria for epilepsy compared with just 8.6% of incident seizure cases that were recorded clinically with epilepsy by the veterinary teams. Furthermore, the relatively small overlap (10.4%) of cases that were both clinically diagnosed with epilepsy and that also received a retrospective classification of epilepsy (idiopathic or structural) indicates substantial discordance and suggests the value of improved education of first opinion practitioners about seizure classification and better dissemination of guidelines^2^, ^4^ to support clinical recording of epilepsy and seizures with more confidence.

Dogs that were <12 years of age or insured had a higher probability of receiving an IVETF Tier 1 or higher diagnostic evaluation. Owners of dogs with seizures might not see the value of an extensive diagnostic evaluation for older dogs, possibly because of concerns about limited survival time and potentially poorer prognosis, especially because structural epilepsy might be more likely at this age.^4^, ^14^, ^15^ Costs of veterinary medical services can rapidly escalate and even a basic clinical evaluation often can exceed the financial limits of many pet owners. Pet insurance may promote access to higher medical standards for companion animals by decreasing financial constraints on diagnostic testing and treatments.^16^, ^17^ The figure of 14.1% insured dogs might be biased because we focused only on the subset of seizure‐affected dogs, but nevertheless seizure cases that were insured were more likely to receive IVETF Tier 1 or higher diagnostic evaluation.

Dogs with a higher body weight compared to their sex and breed mean had a higher probability of a higher diagnostic seizure evaluation. Because dogs with increased body weight were more likely to develop status epilepticus^18^ compared to lower weight dogs, pet owners may be more willing to support more thorough investigation of animals with status epilepticus.^19^

First opinion veterinarians were more likely to diagnose epilepsy when they performed an IVETF Tier 1 or higher diagnostic evaluation with or without MRI and CSF analysis (Tier 2).^4^, ^20^, ^21^ Previous evidence suggests that seizure frequency and intensity influence seizure classification, diagnostic effort and variables considered^19^ by first opinion veterinarians. This phenomenon also may have influenced the probability of an epilepsy diagnosis in our study, but we were unable to assess this impact because data were extracted on seizure count only to classify cases as single seizure or multiple seizure events. A considerable proportion (33.1%) of epilepsy cases diagnosed in first opinion practice received less than a Tier 1 diagnostic effort, suggesting that routine first opinion care does not aspire to, or is not expected to, reach Tier 1 diagnostic depth.

With the help of the IVETF scheme published in 2015, a possible underlying seizure etiology can be tentatively arrived at by considering signalment (eg, age) as well as clinical presentation during or between seizure episodes (eg, idiopathic vs structural epilepsy).^4^ In our study, 26.8% of cases could be classified according to the IVETF guidelines. Our study focused on a time period (2013) before publication of the IVETF guidelines in 2015 and purebred dogs represented the predominant proportion (80.4%) of animals that matched the criteria for idiopathic epilepsy. These findings parallel a previous study that identified 83.6% of idiopathic epileptic dogs as purebred and 16.4% as crossbred dogs.^22^ Because the distribution of purebred, crossbred, and designer dogs was similar in each IVETF category, similar risks for purebreds are likely across the different IVETF categories. The Labrador Retriever, Border Collie, and the Staffordshire Bull Terrier were the 3 most common breeds identified in the idiopathic epilepsy group. This result resembles the findings of a previous study.^23^ In another study, analysis of clinical records from 87 317 dogs indicated that the Labrador Retriever, Parson (Jack) Russell Terrier, and German Shepherd Dog are the breeds with the most frequent epilepsy diagnosis in the United Kingdom.^24^ In a referral population in Tokyo, Japan Chihuahua, Miniature Dachshund, and mixed breed were the 3 most common breeds diagnosed with epilepsy.^21^ Another study from a German referral clinic identified the Border Collie as a breed with a high prevalence of idiopathic epilepsy.^25^

The predominant breed status among the structural epilepsy group in our study was purebred. Breed distribution among dogs with structural epilepsy in our study was affected by the overall breed distribution in the UK dog population, and therefore commonly affected breeds are not necessarily predisposed to structural epilepsy. However, results from the multivariable analysis did take into account the different counts of breeds in the study and therefore provide information on breed predisposition, regardless of the popularity of these breeds overall.^26^ When considering the background population, crossbreds presented the highest number of dogs with structural epilepsy followed by the Labrador Retriever, Staffordshire‐Bull‐Terrier, and Jack Russell Terrier. A study in a referral setting reported crossbreds as being the most common group with structural epilepsy followed by Labrador Retrievers and Boxers.^27^ In addition, mean age at the first seizure event among dogs with structural epilepsy in a previous study was 9.99 years, emphasizing that structural epilepsy is more common in older patients.^4^ Consistent with this finding, dogs ≥9 years of age in our study accounted for 63.1% of structural epilepsy cases. Two retrospective studies support these findings and reported increased age at first seizure onset for dogs with structural epilepsy compared to those with idiopathic epilepsy.^28^, ^29^

The influence of sex and neuter status on seizure activity is the subject of ongoing VetCompass research, with several investigations suggesting a seizure protective effect of androgens,^30^, ^31^ with neutered male dogs reported to have increased risk of developing seizures and epilepsy.^1^, ^23^, ^30^ Our investigation, however, does not support this view because the proportions of neutered dogs both, males and females, were comparable with the wider background population and seizure caseload in our study. Associations between neuter status and outcomes later in life should be considered with caution in cross‐sectional studies because of the potential for reverse causality whereby the outcome (eg, seizures) could have promoted neutering rather than the converse.^32^

Initial treatment of seizure patients aims at terminating seizure activity and preventing further seizure events.^6^ American College of Veterinary Internal Medicine (ACVIM) and IVETF recommend ASD initiation (a) in the presence of a structural lesion or prior brain disease; (b) for acute repetitive seizures or status epilepticus; (c) if ≥2 seizure events occur within a 6‐month period; and, (d) in the presence of prolonged, severe, or unusual postictal periods.^7^, ^33^ The most commonly prescribed ASD in our study were benzodiazepines (12.7%), phenobarbital (9.6%), and imepitoin (2.2%). The dominant role for benzodiazepines is in agreement with previous studies.^34^, ^35^ A high percentage of animals in our study did not receive medical treatment for their first seizure event. The first seizure event often triggers a period of careful observation of the animal for repeated seizure activity. The high frequency of benzodiazepine prescriptions might be a result of pet owners receiving these for use at home in emergency situations. However, in our study, two‐thirds of the dogs with incident seizures did not receive an ASD after the first recorded seizure event, suggesting that UK veterinarians do not routinely start treatment after the first seizure. This observation agrees with the later published IVETF recommendations not to initiate ASD treatment after the incident seizure event.^7^, ^36^

Our study had several limitations, which have been discussed previously.^1^ Retrospective seizure identification is based on the reporting veterinarians notes collected in the VetCompass Program, and cases might be mis‐ or un‐classified because of a potential lack of information because veterinary caregivers write notes for the individual management of each case, and not for research purposes; these records then are adapted by VetCompass as secondary data for research approach. Consequently, these data are expected to be formatted to conform with good clinical practice and professional conduct code,^37^ and to meet the needs of the note‐writing veterinarian to optimize patient care but might not include all data fields or meet levels of data completeness required for research.^38^ Conversely, a surplus of recorded patient information on potential seizure causes might explain the number of unclassifiable cases.

Given the evidential data gap on seizure management for the general dog population, our study provides useful benchmarking information for practitioners on the etiologies, diagnostic evaluation and management of seizures currently used in general practice in the UK^39^ and also provide an evidence‐based context for other studies that report on cases derived from primary care caseloads. Our study analyzed data from 2013, 2 years before IVETF published their recommendations for classification, diagnostic evaluation and treatment.^2^, ^4^, ^7^, ^36^ Future research could repeat the current study to explore the impact of the IVETF on seizure management by first opinion practitioners.

## 5 CONCLUSION

The substantial differences identified in our study between seizure classifications recorded in clinical records and those retrospectively assigned by researchers according to IVETF guidelines support the need for clearer diagnostic guidelines in clinical practice. Insured and younger dogs are more likely to receive advanced diagnostic evaluation, suggesting that financial and perceived prognostic factors influence case management. Our results suggest that animal welfare and diagnostic certainty can be increased by enhanced uptake of pet insurance. The benchmark information provided in our study on the current depth of diagnostic evaluation in the context of etiology and treatment could provide a basis for future studies to assess changes over time after release of the IVETF recommendations for classification, diagnostic evaluation and treatment.

## CONFLICT OF INTEREST DECLARATION

Authors declare no conflict of interest.

## OFF‐LABEL ANTIMICROBIAL DECLARATION

Authors declare no off‐label use of antimicrobials.

## INSTITUTIONAL ANIMAL CARE AND USE COMMITTEE (IACUC) OR OTHER APPROVAL DECLARATION

Authors declare no IACUC or other approval was needed.

## HUMAN ETHICS APPROVAL DECLARATION

Authors declare human ethics approval was not needed for this study.

## Supporting information




**Appendix** **S1**: Supplementary Information.
