# Supplementary material for: Seizures in dogs under primary veterinary care in the United Kingdom: Etiology, diagnostic testing, and clinical management
Source: J Vet Intern Med. 2020 Oct 31;34(6):2525–35. doi: 10.1111/jvim.15911 (PMC7694840; doi:10.1111/jvim.15911)
Supplement: Supplementary file 1 — Appendix S1: Supplementary Information. [file JVIM-34-2525-s001.pdf]

Supplementary Data

Tables and data represent in this file offer an extended access on statistical and analytical results of this recent investigation.

Tables

Table 1: Descriptive statistics of incident seizure cases under primary veterinary care in the UK dog population subdivided in retrospective IVETF Classification, epilepsy diagnosis in first opinion setting and diagnostic evaluation classification

			Retrospective IVETF Classification					Clinically diagnosed with epilepsy in first opinion setting		Diagnostic Work-up		
		All cases No. (%)	Idiopathic Epilepsy No. (%)	Structural Epileptic No. (%)	Reactive Seizure No. (%)	Unclassified No. (%)	No Cause Recorded No. (%)	Epileptic No. (%)	Not Epileptic No. (%)	No. below IVETF Tier 1 Work-up (%)	No. of IVETF Tier 1or higher Work-up (%)	No info
Total		2834 (100.0)	484 (17.1)	95 (3.4)	179 (6.3)	910 (32.1)	1166 (41.1)	245 (8.6%)	2589 (91.4%)	1242 (43.8%	1415 (49.9%	177 (6.2%)
Purebred	Purebred	2240 (79.0)	389 (80.4)	75 (78.9)	144 (80.4)	718 (78.9)	914 (78.4)	54 (22.0)	463 (17.9)	242 (19.5)	237 (16.7)	38 (21.5)
	Crossbred	517 (18.2)	79 (16.3)	20 (21.1)	31 (17.3)	164 (18.0)	223 (19.1)	5 (2.0)	63 (2.4)	24 (1.9)	41 (2.9)	3 (1.7)
	Designer	68 (2.4)	16 (3.3)	0 (0.0)	4 (2.2)	26 (2.9)	22 (1.9)	185 (75.5)	2055 (79.4)	974 (78.4)	1130 (79.9)	136 (76.8)
	Unknown	9 (0.3)	0 (0.0)	0 (0.0)	0 (0.0)	2 (0.2)	7 (0.6)	1 (0.4)	8 (0.3)	2 (0.2)	7 (0.5)	0 (0.0)
Breeds	Crossbred	517 (18.2)	79 (16.3)	20 (21.1)	31 (17.3)	164 (18.0)	223 (19.1)	54 (22.0)	463 (17.9)	242 (19.5)	237 (16.7)	38 (21.5)
	Purebred	339 (12.0)	49 (10.1)	13 (13.7)	28 (15.6)	101 (11.1)	148 (12.7)	34 (13.9)	305 (11.8)	145 (11.7)	169 (11.9)	25 (14.1)
	Labrador Retriever	243 (8.6)	63 (13.0)	6 (6.3)	13 (7.3)	78 (8.6)	83 (7.1)	24 (9.8)	219 (8.5)	97 (7.8)	136 (9.6)	10 (5.6)
	Staffordshire Bull Terrier	173 (6.1)	35 (7.2)	8 (8.4)	8 (4.5)	51 (5.6)	71 (6.1)	10 (4.1)	163 (6.3)	70 (5.6)	95 (6.7)	8 (4.5)
	Jack Russell Terrier	165 (5.8)	23 (4.8)	4 (4.2)	9 (5.0)	57 (6.3)	72 (6.2)	12 (4.9)	153 (5.9)	76 (6.1)	80 (5.7)	9 (5.1)
	Yorkshire Terrier	141 (5.0)	18 (3.7)	4 (4.2)	14 (7.8)	38 (4.2)	67 (5.7)	7 (2.9)	134 (5.2)	64 (5.2)	62 (4.4)	15 (8.5)
	Border Collie	130 (4.6)	36 (7.4)	4 (4.2)	3 (1.7)	37 (4.1)	50 (4.3)	19 (7.8)	111 (4.3)	51 (4.1)	73 (5.2)	6 (3.4)
	Pug	86 (3.0)	19 (3.9)	4 (4.2)	6 (3.4)	29 (3.2)	28 (2.4)	7 (2.9)	79 (3.1)	37 (3.0)	43 (3.0)	6 (3.4)
	Chihuahua	83 (2.9)	14 (2.9)	2 (2.1)	11 (6.1)	23 (2.5)	33 (2.8)	3 (1.2)	80 (3.1)	40 (3.2)	37 (2.6)	6 (3.4)
	German Shepherd Dog	80 (2.8)	15 (3.1)	4 (4.2)	7 (3.9)	26 (2.9)	28 (2.4)	8 (3.3)	72 (2.8)	37 (3.0)	38 (2.7)	5 (2.8)
	Cavalier King Charles Spaniel	78 (2.8)	13 (2.7)	3 (3.2)	3 (1.7)	30 (3.3)	29 (2.5)	6 (2.4)	72 (2.8)	31 (2.5)	46 (3.3)	1 (0.6)
	Border Terrier	75 (2.6)	12 (2.5)	3 (3.2)	3 (1.7)	30 (3.3)	27 (2.3)	3 (1.2)	72 (2.8)	28 (2.3)	45 (3.2)	2 (1.1)
	Boxer	74 (2.6)	6 (1.2)	4 (4.2)	7 (3.9)	25 (2.7)	32 (2.7)	5 (2.0)	69 (2.7)	35 (2.8)	38 (2.7)	1 (0.6)
	West Highland White Terrier	54 (1.9)	2 (0.4)	2 (2.1)	5 (2.8)	16 (1.8)	29 (2.5)	7 (2.9)	47 (1.8)	19 (1.5)	28 (2.0)	7 (4.0)
	Shih-tzu	48 (1.7)	3 (0.6)	2(2.1)	4 (2.2)	13 (1.4)	26 (2.2)	3 (1.2)	45 (1.7)	25 (2.0)	19 (1.3)	4 (2.3)
	Cocker Spaniel	47 (1.7)	7 (1.4)	1 (1.1)	4 (2.2)	16 (1.8)	19 (1.6)	0 (0.0)	47 (1.8)	20 (1.6)	27 (1.9)	0 (0.0)
	Golden Retriever	46 (1.6)	6 (1.2)	1 (1.1)	2 (1.1)	21 (2.3)	16 (1.4)	5 (2.0)	41 (1.6)	21 (1.7)	21 (1.5)	4 (2.3)
	Beagle	32 (1.1)	12 (2.5)	1 (1.1)	0 (0.0)	11 (1.2)	8 (0.7)	4 (1.6)	28 (1.1)	10 (0.8)	17 (1.2)	5 (2.8)
	Springer Spaniel	32 (1.1)	9 (1.9)	1 (1.1)	0 (0.0)	11 (1.2)	11 (0.9)	3 (1.2)	29 (1.1)	18 (1.4)	14 (1.0)	0 (0.0)
	Designer	31 (1.1)	6 (1.2)	0 (0.0)	1 (0.6)	11 (1.2)	13 (1.1)	1 (0.4)	30 (1.2)	11 (0.9)	19 (1.3)	1 (0.6)
	Lhasa Apso	31 (1.1)	4 (0.8)	1 (1.1)	2 (1.1)	10 (1.1)	14 (1.2)	6 (2.4)	25 (1.0)	16 (1.3)	12 (0.8)	3 (1.7)
	Bichon	29 (1.0)	4 (0.8)	1 (1.1)	3 (1.7)	8 (0.9)	13 (1.1)	1 (0.4)	28 (1.1)	16 (1.3)	12 (0.8)	1 (0.6)
	British Bulldog	25 (0.9)	3 (0.6)	0 (0.0)	1 (0.6)	7 (0.8)	14 (1.2)	1 (0.4)	24 (0.9)	13 (1.0)	11 (0.8)	1 (0.6)

	English Springer Spaniel	23 (0.8)	3 (0.6)	0 (0.0)	0 (0.0)	9 (1.0)	11 (0.9)	1 (0.4)	22 (0.8)	8 (0.6)	11 (0.8)	4 (2.3)
	Miniature Schnauzer	22 (0.8)	0 (0.0)	1 (1.1)	2 (1.1)	6 (0.7)	13 (1.1)	0 (0.0)	22 (0.8)	12 (1.0)	9 (0.6)	1 (0.6)
	Lurcher	19 (0.7)	4 (0.8)	0 (0.0)	2 (1.1)	8 (0.9)	5 (0.4)	1 (0.4)	18 (0.7)	8 (0.6)	10 (0.7)	1 (0.6)
	Pomeranian	19 (0.7)	4 (0.8)	1 (1.1)	2 (1.1)	6 (0.7)	6 (0.5)	1 (0.4)	18 (0.7)	7 (0.6)	10 (0.7)	2 (1.1)
	Husky	18 (0.6)	8 (1.7)	0 (0.0)	1 (0.6)	3 (0.3)	6 (0.5)	3 (1.2)	15 (0.6)	7 (0.6)	10 (0.7)	1 (0.6)
	Labradoodle	18 (0.6)	6 (1.2)	0 (0.0)	1 (0.6)	7 (0.8)	4 (0.3)	3 (1.2)	15 (0.6)	5 (0.4)	12 (0.8)	1 (0.6)
	Patterdale Terrier	18 (0.6)	5 (1.0)	1 (1.1)	0 (0.0)	8 (0.9)	4 (0.3)	0 (0.0)	18 (0.7)	9 (0.7)	9 (0.6)	0 (0.0)
	Dogue de Bordeaux	17 (0.6)	4 (0.8)	1 (1.1)	0 (0.0)	5 (0.5)	7 (0.6)	2 (0.8)	15 (0.6)	6 (0.5)	10 (0.7)	1 (0.6)
	Miniature Dachshund	16 (0.6)	3 (0.6)	0 (0.0)	2 (1.1)	4 (0.4)	7 (0.6)	1 (0.4)	15 (0.6)	7 (0.6)	9 (0.6)	0 (0.0)
	King Charles Spaniel	16 (0.6)	3 (0.6)	0 (0.0)	0 (0.0)	6 (0.7)	7 (0.6)	2 (0.8)	14 (0.5)	6 (0.5)	9 (0.6)	1 (0.6)
	Basset Hound	14 (0.5)	1 (0.2)	0 (0.0)	1 (0.6)	5 (0.5)	7 (0.6)	0 (0.0)	14 (0.5)	10 (0.8)	2 (0.1)	2 (1.1)
	PoodlToy	14 (0.5)	0 (0.0)	0 (0.0)	0 (0.0)	9 (1.0)	5 (0.4)	0 (0.0)	14 (0.5)	10 (0.8)	4 (0.3)	0 (0.0)
	Rottweiler	14 (0.5)	2 (0.4)	0 (0.0)	1 (0.6)	2 (0.2)	9 (0.8)	1 (0.4)	13 (0.5)	5 (0.4)	6 (0.4)	3 (1.7)
	Akita	13 (0.5)	1 (0.2)	1 (1.1)	1 (0.6)	5 (0.5)	5 (0.4)	4 (1.6)	9 (0.3)	7 (0.6)	5 (0.4)	1 (0.6)
	Weimaraner	13 (0.5)	0 (0.0)	1 (1.1)	1 (0.6)	7 (0.8)	4 (0.3)	0 (0.0)	13 (0.5)	6 (0.5)	7 (0.5)	0 (0.0)
	French Bulldog	12 (0.4)	2 (0.4)	0 (0.0)	0 (0.0)	5 (0.5)	5 (0.4)	2 (0.8)	10 (0.4)	5 (0.4)	6 (0.4)	1 (0.6)
	Unknown	9 (0.3)	0 (0.0)	0 (0.0)	0 (0.0)	2 (0.2)	7 (0.6)	1 (0.4)	8 (0.3)	2 (0.2)	7 (0.5)	0 (0.0)
KC Breed Group	Not KC recognized	820 (28.9)	131 (27.1)	27 (28.4)	46 (25.7)	268 (29.5)	348 (29.8)	76 (31.0)	744 (28.7)	374 (30.1)	394 (27.8)	52 (29.4)
	Gundog	455 (16.1)	99 (20.5)	10 (10.5)	22 (12.3)	159 (17.5)	165 (14.2)	38 (15.5)	417 (16.1)	192 (15.5)	240 (17.0)	23 (13.0)
	Hound	94 (3.3)	21 (4.3)	1 (1.1)	7 (3.9)	29 (3.2)	36 (3.1)	9 (3.7)	85 (3.3)	37 (3.0)	48 (3.4)	9 (5.1)
	Pastoral	241 (8.5)	57 (11.8)	8 (8.4)	12 (6.7)	71 (7.8)	93 (8.0)	28 (11.4)	213 (8.2)	96 (7.7)	130 (9.2)	15 (8.5)
	Terrier	363 (12.8)	56 (11.6)	17 (17.9)	19 (10.6)	116 (12.7)	155 (13.3)	28 (11.4)	335 (12.9)	141 (11.4)	201 (14.2)	21 (11.9)
	Toy	484 (17.1)	78 (16.1)	16 (16.8)	43 (24.0)	149 (16.4)	198 (17.0)	31 (12.7)	453 (17.5)	214 (17.2)	233 (16.5)	37 (20.9)
	Utility	235 (8.3)	23 (4.8)	8 (8.4)	18 (10.1)	73 (8.0)	113 (9.7)	19 (7.8)	216 (8.3)	125 (10.1)	97 (6.9)	13 (7.3)
	Working	142 (5.0)	19 (3.9)	8 (8.4)	12 (6.7)	45 (4.9)	58 (5.0)	16 (6.5)	126 (4.9)	63 (5.1)	72 (5.1)	7 (4.0)
Adult Bodyweight (kg)	< 10.00	673 (23.7)	113 (23.3)	17 (17.9)	58 (32.4)	206 (22.6)	279 (23.9)	50 (20.4)	623 (24.1)	307 (24.7)	334 (23.6)	32 (18.1)
	10.00 - ≤ 20.00	449 (15.8)	103 (21.3)	21 (22.1)	19 (10.6)	128 (14.1)	178 (15.3)	57 (23.3)	392 (15.1)	179 (14.4)	250 (17.7)	20 (11.3)
	20.00 - ≤ 30.00	348 (12.3)	86 (17.8)	7 (7.4)	11 (6.1)	117 (12.9)	127 (10.9)	33 (13.5)	315 (12.2)	148 (11.9)	191 (13.5)	9 (5.1)
	30.00 - ≤ 40.00	240 (8.5)	54 (11.2)	6 (6.3)	15 (8.4)	61 (6.7)	104 (8.9)	29 (11.8)	211 (8.1)	103 (8.3)	123 (8.7)	14 (7.9)
	≥ 40.00	77 (2.7)	12 (2.5)	5 (5.3)	5 (2.8)	34 (3.7)	21 (1.8)	5 (2.0)	72 (2.8)	31 (2.5)	44 (3.1)	2 (1.1)
	unrecorded	1047 (36.9)	116 (24.0)	39 (41.1)	71 (39.7)	364 (40.0)	457 (39.2)	71 (29.0)	976 (37.7)	474 (38.2)	473 (33.4)	100 (56.5)
		median (IQR)	median (IQR)	median (IQR)	median (IQR)	median (IQR)	median (IQR)	median (IQR)	median (IQR)	median (IQR)	median (IQR)	median (IQR)
	Median adult Bodyweight (kg)	16.12 (8.33-28.04)	19.03 (9.34-29.31)	16.15 (9.00-28.50)	10.80 (6.65-27.86)	16.01 (8.43-28.58)	15.34 (7.89-26.56)	17.60 (9.88-29.28)	15.58 (8.13-27.87)	15.23 (7.80-26.86)	16.88 (8.88-28.84)	15.39 (7.53-25.44)
Adult Bodyweight relative to breed and sex mean	Lower	1265 (44.6)	211 (43.6)	41 (43.2)	78 (43.6)	435 (47.8)	500 (42.9)	122 (49.8)	1143 (44.1)	583 (46.9)	618 (43.7)	64 (36.2)
	Equal/Higher	1129 (39.8)	249 (51.4)	36 (37.9)	56 (31.3)	360 (39.6)	428 (36.7)	95 (38.8)	1034 (39.9)	437 (35.2)	634 (44.8)	58 (32.8)
	unrecorded	440 (15.5)	24 (5.0)	18 (18.9)	45 (25.1)	115 (12.6)	238 (20.4)	28 (11.4)	412 (15.9)	222 (17.9)	163 (11.5)	55 (31.1)
Age at first Seizure (years)	0.00 - ≤ 0.50	91 (3.2)	4 (0.8)	3 (3.2)	18 (10.1)	26 (2.9)	40 (3.4)	3 (1.2)	88 (3.4)	43 (3.5)	43 (3.0)	5 (2.8)
	0.50 - ≤ 3.00	622 (21.9)	229 (47.3)	6 (6.3)	30 (16.8)	188 (20.7)	169 (14.5)	64 (26.1)	558 (21.6)	225 (18.1)	372 (26.3)	25 (14.1)
	3.00 - ≤ 6.00	728 (25.7)	251 (51.9)	8 (8.4)	31 (17.3)	203 (22.3)	235 (20.2)	59 (24.1)	669 (25.8)	303 (24.4)	396 (28.0)	29 (16.4)
	6.00 - ≤ 9.00	533 (18.8)	0 (0.0)	18 (18.9)	28 (15.6)	230 (25.3)	257 (22.0)	45 (18.4)	488 (18.8)	235 (18.9)	263 (18.6)	35 (19.8)
	9.00 - ≤ 12.00	397 (14.0)	0 (0.0)	31 (32.6)	37 (20.7)	141 (15.5)	188 (16.1)	37 (15.1)	360 (13.9)	183 (14.7)	185 (13.1)	29 (16.4)
	≥ 12.00	444 (15.7)	0 (0.0)	29 (30.5)	31 (17.3)	116 (12.7)	268 (23.0)	36 (14.7)	408 (15.8)	246 (19.8)	147 (10.4)	51 (28.8)
	Unrecorded	19 (0.7)	0 (0.0)	0 (0.0)	4 (2.2)	6 (0.7)	9 (0.8)	1 (0.4)	18 (0.7)	7 (0.6)	9 (0.6)	3 (1.7)

		median (IQR)	median (IQR)	median (IQR)	median (IQR)	median (IQR)	median (IQR)	median (IQR)	median (IQR)	median (IQR)	median (IQR)	media n (IQR)
	Median Age at first seizure	6.00 (3.00-10.10)	3.15 (2.15-4.40)	11.00 (7.75-13.20)	6.80 (3.00-11.30)	6.50 (3.25-9.70)	7.70 (4.00-11.90)	5.75 (3.00-10.45)	6.00 (3.00-10.10)	6.60 (3.50-8.90)	5.10 (2.80-8.90)	8.45 (4.90-12.50)
Sex	Female	1240 (43.8)	152 (31.4)	47 (49.5)	103 (57.5)	392 (43.1)	546 (46.8)	111 (45.3)	1129 (43.6)	556 (44.8)	594 (42.0)	90 (50.8)
	Male	1587 (56.0)	331 (68.4)	48 (50.5)	76 (42.5)	517 (56.8)	615 (52.7)	133 (54.3)	1454 (56.2)	683 (55.0)	817 (57.7)	87 (49.2)
	Unrecorded	7 (0.2)	1 (0.2)	0 (0.0)	0 (0.0)	1 (0.1)	5 (0.4)	1 (0.4)	6 (0.2)	3 (0.2)	4 (0.3)	0 (0.0)
Neuter status	Entire	891 (31.4)	149 (30.8)	29 (30.5)	63 (35.2)	271 (29.8)	379 (32.5)	89 (36.6)	802 (31.0)	398 (32.0)	433 (30.6)	60 (33.9)
	Neutered	1303 (46.0)	262 (54.1)	46 (48.4)	78 (43.6)	403 (44.3)	514 (44.1)	112 (45.7)	1191 (46.0)	556 (44.8)	681 (48.1)	66 (37.3)
	Unknown	640 (22.6)	73 (15.1)	20 (21.1)	38 (21.2)	236 (25.9)	273 (23.4)	44 (18.0)	596 (23.0)	288 (23.2)	301 (21.3)	51 (28.8)
Sex-neuter	Female/Entire	335 (11.8)	37 (7.6)	11 (11.6)	31 (17.3)	103 (11.3)	153 (13.1)	36 (14.7)	229 (11.5)	155 (12.5)	153 (10.8)	27 (15.3)
	Female/Neutered	625 (22.1)	96 (19.8)	24 (25.3)	51 (28.5)	184 (20.2)	270 (23.2)	51 (20.8)	574 (22.2)	271 (21.8)	311 (22.0)	43 (24.3)
	Female/unrecorded	280 (9.9)	19 (3.9)	12 (12.6)	21 (11.7)	105 (11.5)	123 (10.5)	24 (9.8)	256 (9.9)	130 (10.5)	130 (9.2)	20 (11.3)
	Male/Entire	550 (19.4)	111 (22.9)	18 (18.9)	32 (17.9)	167 (18.4)	222 (19.0)	52 (21.2)	498 (19.2)	240 (19.3)	277 (19.6)	33 (18.6)
	Male/Neutered	677 (23.9)	166 (34.3)	22 (23.2)	27 (15.1)	219 (24.1)	243 (20.8)	61 (24.9)	616 (23.8)	285 (22.9)	369 (26.1)	23 (13.0)
	Male/unrecorded	360 (12.7)	54 (11.2)	8 (8.4)	17 (9.5)	131 (14.4)	150 (12.9)	20 (8.2)	340 (13.1)	158 (12.7)	171 (12.1)	31 (17.5)
	unrecorded/unrecorded	7 (0.2)	1 (0.2)	0 (0.0)	0 (0.0)	1 (0.1)	5 (0.4)	1 (0.4)	6 (0.2)	3 (0.2)	4 (0.3)	0 (0.0)
Insurance Status	Iinsured	521 (18.4)	137 (28.3)	13 (13.7)	28 (15.6)	190 (20.9)	153 (13.1)	10 (4.1)	207 (8.0)	175 (14.1)	320 (22.6)	26 (14.7)
	Not insured	217 (7.7)	26 (5.4)	7 (7.4)	11 (6.1)	81 (8.9)	92 (7.9)	44 (18.0)	477 (18.4)	100 (8.1)	97 (6.9)	20 (11.3)
	unrecorded	2096 (74.0)	321 (66.3)	75 (78.9)	140 (78.2)	639 (70.2)	921 (79.0)	191 (78.0)	1905 (73.6)	967 (77.9)	998 (70.5)	131 (74.0)
Diagnostic Work-up	below IVETF Tier 1	1242 (43.8)	0 (0.0)	48 (50.5)	67 (37.4)	445 (48.9)	682 (58.5)	81 (33.1)	1161 (44.8)			
	IVETF Tier 1or higher	1415 (49.9)	484 (100.0)	47 (49.5)	103 (57.5)	439 (48.2)	342 (29.3)	154 (62.9)	1261 (48.7)			
	unrecorded	177 (6.2)	0 (0.0)	0 (0.0)	9 (5.0)	26 (2.9)	142 (12.2)	10 (4.1)	167 (6.5)			
	MRI	80 (2.8)	18 (3.7)	1 (1.1)	2 (1.1)	32 (3.5)	27 (2.3)	34 (13.9)	46 (1.8)	30 (2.4)	48 (3.4)	2 (1.1)
	CSF	47 (1.7)	8 (1.7)	1 (1.1)	1 (0.6)	20 (2.2)	17 (1.5)	23 (9.4)	24 (0.9)	19 (1.5)	27 (1.9)	1 (0.6)
Clinically recoded	Not epileptic	2589 (91.4)								1161 (93.5)	1261 (89.1)	167 (94.4)
	epileptic	245 (8.6)								81 (6.5)	154 (10.9)	10 (5.6)

Table 1: Descriptive statistic results among incident seizure cases in the UK dog population under primary veterinary care; etiologic classification, clinical diagnosis with epilepsy in first opinion setting and diagnostic evaluation

Table 2: Final breed multivariable logistic regression results for risk factors associated with clinical diagnosis with epilepsy in first opinion setting from all incident seizure cases under primary veterinary care in the UK dog population

Variable	Category	Odds ratio	95% CIa		Category P-value	Variable P-value
Breeds	Labrador Retriever	Base				P = 0.495
	Akita	6.01	1.59	22.82	0.008	
	Beagle	2.07	0.65	6.60	0.22	
	Bichon	0.33	0.04	2.83	0.31	
	Boxer	0.70	0.24	2.00	0.50	
	British Bulldog	0.58	0.073	4.56	0.60	
	French Bulldog	2.32	0.44	12.37	0.32	
	Chihuahua	0.41	0.11	1.47	0.171	
	Cavalier King Charles Spaniel	1.12	0.43	2.91	0.824	
	Border Collie	1.99	1.00	3.96	0.050	
	Crossbred	1.45	0.83	2.51	0.189	
	Miniature Dachshund	0.44	0.04	4.55	0.487	
	Dogue de Bordeaux	1.87	0.39	8.98	0.435	
	Designer	0.44	0.05	3.49	0.433	
	English Springer Spaniel	0.73	0.09	5.80	0.768	
	German Shepherd Dog	1.36	0.55	3.34	0.507	
	Basset Hound	0.00	0.00		0.999	
	Husky	2.79	0.72	10.78	0.138	
	Jack Russell Terrier	1.01	0.47	2.15	0.990	
	Labradoodle	1.56	0.37	6.56	0.541	
	Lhasa Apso	2.96	1.02	8.56	0.046	
	Lurcher	0.76	0.10	6.07	0.794	
	Pomeranian	0.50	0.05	4.77	0.549	
	Toy Poodle	0.00	0.00		0.999	
	Pug	0.99	0.39	2.53	0.989	
	Purebred	1.42	0.79	2.57	0.240	
	Golden Retriever	1.80	0.63	5.15	0.276	
	Rottweiler	1.05	0.13	8.62	0.963	
	Miniature Schnauzer	0.00	0.00	0.00	0.998	
	Shih-tzu	0.68	0.17	2.64	0.575	
	King Charles Spaniel	2.05	0.42	10.02	0.376	
	Cocker Spaniel	0.00	0.00	0.00	0.997	
	Springer Spaniel	0.95	0.25	3.58	0.938	
	Patterdale Terrier	0.00	0.00	0.00	0.998	
	Border Terrier	0.50	0.14	1.74	0.275	
	Staffordshire Bull Terrier	0.69	0.31	1.55	0.372	
	Unknown	1.60	0.17	14.97	0.682	
	Weimaraner	0.00	0.00	0.00	0.999	
	West Highland White Terrier	1.56	0.60	4.06	0.365	
	Yorkshire Terrier	0.68	0.28	1.67	0.398	
Bodyweight relative to breed mean	Lower	Base				P = 0.117
	Equal/Higher	0.78	0.58	1.05	0.098	
	unrecorded	0.68	0.43	1.08	0.101	
Age at first Seizure (years)						P = 0.444

	0.00 - ≤ 0.50	0.33	0.09	1.18	0.089	
	0.50 - ≤ 3.00	1.02	0.64	1.65	0.922	
	3.00 - ≤ 6.00	0.80	0.49	1.28	0.345	
	6.00 - ≤ 9.00	0.94	0.58	1.55	0.820	
	9.00 - ≤ 12.00	1.13	0.67	1.88	0.652	
	> 12.00	Base				
	unrecorded	0.58	0.07	5.08	0.622	
Sex-neuter	Female/Entire	Base				P = 0.524
	Female/Neutered	0.63	0.39	1.03	0.067	
	Female/unrecorded	0.82	0.45	1.48	0.502	
	Male/Entire	0.82	0.51	1.32	0.411	
	Male/Neutered	0.75	0.47	1.21	0.237	
	Male/unrecorded	0.58	0.31	1.08	0.086	
	unrecorded/unrecorded	1.59	0.16	15.85	0.695	
Insurance Status						P = 0.142
	is insured	1.81	0.86	3.83	0.120	
	not insured	Base				
	unrecorded	2.03	1.00	4.12	0.050	
IVETF Tier 1	below IVETF Tier 1	Base				P < 0.001
	IVETF Tier 1or higher	1.74	1.30	2.34	< 0.001	
	unrecorded	0.89	0.44	1.78	0.733	
MRI	No MRI	Base				P < 0.001
	MRI	6.19	3.03	12.64	< 0.001	
CSF	No CSF	Base				P < 0.001
	CSF	2.80	1.13	6.94	0.027	

Table 2: Risk factors for clinical diagnosis with epilepsy in first opinion setting from incident seizure cases under primary veterinary care in the UK dog population. Base = comparison group

Table 2a: Multivariable logistic regression results for variables that replaced breed

Variable	Category	Odds ratio	95% CIa		Category P-value	Variable P-value
Purebred	Crossbred	Base				P = 0.290
	Designer	0.61	0.22	1.65	0.326	
	Purebred	0.73	0.52	1.02	0.064	
	Unknown	1.08	0.12	9.73	0.948	
KC Breed Group	Not KC recognized	Base				P = 0.293
	Gundog	0.79	0.51	1.22	0.288	
	Hound	1.10	0.52	2.34	0.802	
	Pastoral	1.283	0.79	2.08	0.311	
	Terrier	0.79	0.49	1.25	0.313	
	Toy	0.68	0.43	1.06	0.088	
	Utility	0.85	0.49	1.46	0.553	
	Working	1.27	0.67	2.31	0.439	
Bodyweight (kg)	< 10.00	Base				P = 0.148
	10.00 - ≤ 20.00	1.84	1.18	2.87	0.007	
	20.00 - ≤ 30.00	1.32	0.81	2.16	0.263	
	30.00 - ≤ 40.00	1.38	0.82	2.35	0.228	
	≥ 40.00	1.17	0.51	2.69	0.708	
	unrecorded	1.16	0.72	1.85	0.550	

Table 2a: Results for variables that replaced the breed variable in the final breed multivariable logistic regression model (with Age at first seizure, bodyweight relative to breed mean, Sex-neuter, Insurance status, IVETF Tier 1, MRI, CSF) to evaluate risk factors associated with clinical diagnosis of epilepsy in first opinion setting from incident seizure cases under primary veterinary care in the UK dog population. Bodyweight relative to breed mean was also removed for Bodyweight, aCI confidence interval. Base = comparison group

Table 3: Multivariable logistic regressions results: Risk factors for receiving IVETF Tier 1 or higher diagnostic evaluation

Variable	Category	Odds ratio	95% CIa		Category P-value	Variable P-value
Breeds	Labrador Retriever	Base				P = 0.822
	Akita	0.57	0.17	1.96	0.373	
	Beagle	1.33	0.57	3.13	0.514	
	Bichon	0.61	0.27	1.38	0.231	
	Boxer	0.83	0.48	1.43	0.498	
	British Bulldog	0.52	0.22	1.24	0.142	
	French Bulldog	0.87	0.25	3.06	0.829	
	Chihuahua	0.77	0.45	1.32	0.346	
	Cavalier King Charles Spaniel	1.18	0.69	2.02	0.547	
	Border Collie	1.10	0.70	1.74	0.687	
	Crossbred	0.82	0.59	1.14	0.246	
	Miniature Dachshund	1.06	0.37	3.03	0.908	
	Dogue de Bordeaux	1.23	0.43	3.56	0.699	
	Designer	1.11	0.49	2.50	0.796	
	English Springer Spaniel	0.97	0.37	2.55	0.946	
	German Shepherd Dog	0.85	0.50	1.46	0.566	
	Basset Hound	0.14	0.03	0.70	0.016	
	Husky	0.93	0.33	2.64	0.887	
	Jack Russell Terrier	0.96	0.63	1.46	0.843	
	Labradoodle	1.46	0.48	4.40	0.503	
	Lhasa Apso	0.66	0.29	1.50	0.327	
	Lurcher	1.00	0.36	2.73	0.995	
	Pomeranian	1.11	0.39	3.11	0.847	
	Toy Poodle	0.34	0.10	1.15	0.083	
	Pug	0.76	0.45	1.29	0.314	
	Purebred	0.93	0.66	1.33	0.697	
	Golden Retriever	0.72	0.36	1.41	0.338	
	Rottweiler	0.84	0.25	2.89	0.788	
	Miniature Schnauzer	0.66	0.26	1.66	0.375	
	Shih-tzu	0.75	0.38	1.47	0.407	
	King Charles Spaniel	0.90	0.30	2.72	0.854	
	Cocker Spaniel	1.11	0.58	2.13	0.747	
	Springer Spaniel	0.69	0.32	1.50	0.353	
	Patterdale Terrier	0.80	0.30	2.17	0.667	
	Border Terrier	1.28	0.74	2.21	0.387	
	Staffordshire Bull Terrier	1.13	0.75	1.71	0.566	
	Unknown	3.63	0.69	18.99	0.127	
	Weimaraner	0.94	0.30	2.96	0.917	
	West Highland White Terrier	1.44	0.74	2.79	0.284	
	Yorkshire Terrier	0.89	0.57	1.39	0.608	
Bodyweight relative to breed mean	Lower	Base				P < 0.001
	Equal/Higher	1.37	1.15	1.63	< 0.001	
	Unrecorded	0.72	0.55	0.94	0.015	
Age at first seizure (years)	0.00 - ≤ 0.50	2.50	1.50	4.18	< 0.001	P < 0.001
	0.50 - ≤ 3.00	2.87	2.16	3.81	< 0.001	

	3.00 - ≤ 6.00	2.01	1.54	2.63	< 0.001	
	6.00 - ≤ 9.00	1.74	1.31	2.31	< 0.001	
	9.00 - ≤ 12.00	1.67	1.23	2.26	0.001	
	≥ 12.00	Base				
	Unrecorded	2.60	0.90	7.56	0.079	
Sex-neuter	Female/Entire	Base				P = 0.898
	Female/Neutered	1.14	0.85	1.53	0.392	
	Female/unrecorded	0.99	0.70	1.42	0.973	
	Male/Entire	1.13	0.84	1.52	0.657	
	Male/Neutered	1.07	0.80	1.43	0.657	
	Male/unrecorded	0.96	0.68	1.35	0.815	
	unrecorded/unrecorded	1.26	0.24	6.73	0.786	
Insurance Status	is insured	1.50	1.06	2.13	0.024	P < 0.001
	not insured	Base				
	Unrecorded	0.92	0.67	1.28	0.630	
Clinically recoded with epilepsy	Not epileptic	Base				P < 0.001
	Epileptic	1.74	1.30	2.32	< 0.001	

Table 3: Risk factors for receiving an IVETF Tier 1or higher diagnostic evaluation among incident seizure cases under primary veterinary care in the UK dog population. Base = comparison group

Table 3a: Multivariable logistic regression results for variables that replaced breed

Variable	Category	Odds ratio	95% CIa		Category P-value	Variable P-value
Purebred	Crossbred	Base				P = 0.178
	Designer	1.41	0.81	2.45	0.229	
	Purebred	1.13	0.92	1.39	0.252	
	Unknown	4.40	0.85	22.63	0.077	
KC Breed Group	Not KC recognized	Base				P = 0.021
	Gundog	1.06	0.83	1.35	0.656	
	Hound	1.15	0.72	1.84	0.555	
	Pastoral	1.24	0.91	1.69	0.176	
	Terrier	1.40	1.07	1.83	0.013	
	Toy	0.99	0.78	1.26	0.940	
	Utility	0.72	0.53	0.98	0.037	
	Working	0.94	0.64	1.37	0.741	
Bodyweight (kg)	< 10.00	Base				P = 0.134
	10.00 - ≤ 20.00	1.21	0.93	1.58	0.162	
	20.00 - ≤ 30.00	1.15	0.87	1.52	0.328	
	30.00 - ≤ 40.00	1.11	0.81	1.51	0.512	
	≥ 40.00	1.35	0.84	2.17	0.215	
	Unrecorded	0.86	0.67	1.11	0.252	

Tab 3a: Results for variables that replaced the breed variable in the final breed multivariable logistic regression model (with Bodyweight relative to breed mean, Age at first seizure, Sex-neuter, Insurance status, clinically recorded with epilepsy) to evaluate risk factors associated with receiving IVETF Tier 1 or higher diagnostic work-up from incident seizure cases under primary veterinary care in the UK dog population. Bodyweight relative to breed mean was also removed for Bodyweight, aCI confidence interval. Base = comparison group

Table 4: Medication in association with the first seizure incident of seizure cases under primary veterinary care in the UK dog population subdivided in retrospective IVETF Classification, epilepsy diagnosis in first opinion setting and diagnostic evaluation classification

			Retrospective IVETF Classification						Clinically diagnosed with epilepsy in first opinion setting			Diagnostic Work-up		
	Substance	All cases No (%)	Idiopathic Epilepsy (%)	Structural Epileptic No. (%)	Reactive Seizure No. (%)	Unclassified No. (%)	No Cause Recorded No. (%)	P-value comparing usage for each category between groups	Epileptic first opinion No. (%)	Not Epileptic first opinion No. (%)	P-value comparing usage for each category between groups	No. below IVETF Tier 1Work-up (%)	No. of IVETF Tier 1or higher Work-up (%)	NO INFO about diagnostic work-up (%)
	Received a medication	874 (30.8)	141 (29.1)	23 (24.2)	51 (28.5)	301 (33.1)	358 (30.7)	0.265	148 (60.4)	726 (28.0)	< 0.001	370 (29.8)	451 (31.9)	53 (30.0)
	Received NO medication	1960 (69.2)	343 (70.9)	72 (75.8)	128 (71.5)	609 (66.9)	808 (69.3)		97 (39.6)	1863 (72.0)		872 (70.2)	964 (68.1)	124 (70.0)
	Single Substance treatment	719 (25.3)	111 (22.9)	19 (20.0)	41 (22.9)	249 (27.4)	299 (25.6)		120 (49.0)	599 (23.1)	0.915	306 (24.6)	368 (26.0)	45 (25.4)
	ASD Substance Combination	155 (5.5)	30 (6.2)	4 (4.2)	10 (5.6)	52 (5.7)	59 (5.1)		28 (11.4)	127 (4.9)	< 0.001	64 (5.2)	83 (5.9)	8 (4.6)
ASD Single Substance treatment	Benzodiazepine	360 (12.7)	46 (9.5)	10 (10.5)	17 (9.5)	127 (14.0)	160 (13.7)		31 (12.5)	329 (12.7)	0.915	172 (13.9)	161 (11.4)	27 (14.8)
	Phenobarbital	271 (9.6)	51 (10.5)	8 (8.4)	19 (10.6)	82 (9.0)	111 (9.5)		64 (25.8)	207 (8.0)	< 0.001	109 (8.8)	150 (10.6)	12 (6.6)
	Imepitoin	61 (2.2)	8 (1.7)	0 (0.0)	3 (1.7)	26 (2.6)	24 (2.1)		20 (8.1)	41 (1.6)	< 0.001	29 (2.3)	28 (2.0)	4 (2.2)
	Levetiracetam	10 (0.4)	3 (0.6)	0 (0.0)	1 (0.6)	5 (0.5)	1 (0.1)		3 (1.2)	7 (0.3)	0.147	1 (0.1)	8 (0.6)	1 (0.5)
	Gabapentin	11 (0.4)	3 (0.6)	0 (0.0)	1 (0.6)	5 (0.5)	2 (0.2)		2 (0.8)	9 (0.3)	0.384	5 (0.4)	5 (0.4)	1 (0.5)
	Potassium Bromide	6 (0.2)	0 (0.0)	1 (1.1)	0 (0.0)	4 (0.4)	1 (0.1)		3 (1.2)	3 (0.1)	0.003	0 (0.0)	4 (0.3)	2 (1.1)
	ASD Substance Combination	155 (5.5)	30 (6.2)	4 (4.2)	10 (5.6)	52 (5.7)	59 (5.1)		28 (11.3)	127 (4.9)	< 0.001	64 (5.2)	83 (5.9)	8 (4.4)
	Received No Medication	1960 (69.2)	343 (70.9)	72 (75.8)	128 (71.5)	609 (66.9)	808 (69.3)		97 (39.1)	1863 (72.0)	< 0.001	856 (69.3)	976 (69.0)	128 (69.9)
AED Substance-combination treatment	Benzodiazepine+Phenobarbital	91 (3.2)	18 (3.7)	2 (2.1)	6 (3.4)	26 (2.9)	36 (3.1)		10 (4.1)	78 (3.0)	0.117	36 (3.0)	50 (3.5)	2 (1.1)
	Phenobarbital+Potassium Bromide	18 (0.6)	5 (1.0)	0 (0.0)	0 (0.0)	10 (1.1)	4 (0.3)		6 (2.5)	13 (0.5)	< 0.001	9 (0.7)	10 (0.7)	0 (0.0)

	Benzodiazepine+Imepitoin	16 (0.6)	1 (0.2)	1 (1.1)	1 (0.6)	5 (0.5)	8 (0.7)		4 (1.6)	12 (0.5)	0.020	9 (0.7)	6 (0.4)	1 (0.6)
	Benzodiazepine+Propofol	9 (0.3)	1 (0.2)	0 (0.0)	2 (1.1)	4 (0.4)	2 (0.2)		0 (0.0)	9 (0.3)	0.355	6 (0.5)	3 (0.2)	0 (0.0)
	Benzodiazepine+Levetiracetam	3 (0.1)	0 (0.0)	0 (0.0)	0 (0.0)	3 (0.3)	2 (0.2)		2 (0.8)	3 (0.1)	0.128	4 (0.3)	1 (0.1)	0 (0.0)
	Phenobarbital+Potassiumbromide+Levetiracetam	3 (0.1)	2 (0.4)	0 (0.0)	0 (0.0)	0 (0.0)	1 (0.1)		3 (1.2)	0 (0.0)	< 0.001	0 (0.0)	3 (0.2)	0 (0.0)
	Other	15 (0.5)	3 (0.6)	1 (1.1)	1 (0.6)	4 (0.4)	6 (0.5)		2 (0.8)	13 (0.5)	0.517	4 (0.3)	10 (0.7)	1 (0.6)
	Single Substance treatment	719 (25.4)	111 (22.9)	19 (20.0)	41 (22.9)	249 (27.4)	299 (25.6)		120 (49.2)	599 (23.1)	0.915	306 (24.9)	368 (25.8)	45 (25.4)
	Received No Medication	1960 (69.2)	343 (70.9)	72 (75.8)	128 (71.5)	609 (66.9)	808 (69.3)		97 (39.8)	1863 (72.0)	< 0.001	856 (69.6)	976 (68.4)	128 (72.3)

Table 4: descriptive results for the medical management of incident seizure cases under primary veterinary care in the UK dog population.
